# Metallic 4D Printing of Laser Stimulation

**DOI:** 10.1002/advs.202206486

**Published:** 2023-01-22

**Authors:** Wenzheng Wu, Yiming Zhou, Qingping Liu, Luquan Ren, Fan Chen, Jerry Ying Hsi Fuh, Aodu Zheng, Xuechao Li, Ji Zhao, Guiwei Li

**Affiliations:** ^1^ School of Mechanical and Aerospace Engineering Jilin University Changchun Jilin 130025 P. R. China; ^2^ Key Laboratory of Bionic Engineering (Ministry of Education) Jilin University Changchun 130025 P. R. China; ^3^ Department of Mechanical Engineering National University of Singapore Singapore 117576 Singapore; ^4^ Chongqing Research Institute Jilin University 618 Liangjiang Avenue, Longxing Town, Yubei District Chongqing 401122 P. R. China; ^5^ School of Mechanical Engineering and Automation Northeastern University Shenyang Liaoning 110004 P. R. China

**Keywords:** 4D printing, additive manufacturing, laser powder bed fusion, laser stimulation, metallic shape‐morphing structures

## Abstract

4D printing of metallic shape‐morphing systems can be applied in many fields, including aerospace, smart manufacturing, naval equipment, and biomedical engineering. The existing forming materials for metallic 4D printing are still very limited except shape memory alloys. Herein, a 4D printing method to endow non‐shape‐memory metallic materials with active properties is presented, which could overcome the shape‐forming limitation of traditional material processing technologies. The thermal stress spatial control of 316L stainless steel forming parts is achieved by programming the processing parameters during a laser powder bed fusion (LPBF) process. The printed parts can realize the shape changing of selected areas during or after forming process owing to stress release generated. It is demonstrated that complex metallic shape‐morphing structures can be manufactured by this method. The principles of printing parameters programmed and thermal stress pre‐set are also applicable to other thermoforming materials and additive manufacturing processes, which can expand not only the materials used for 4D printing but also the applications of 4D printing technologies.

## Graphical abstract

A 4D printing method to endow non‐shape‐memory metal materials with programmable shape‐morphing behaviors via pre‐stress assemblies enabled by laser powder bed fusion is reported. The printed parts can realize the shape changing of selected areas during or after forming process owing to stress release generated. The methodology opens new avenues to create metallic shape‐morphing 3D structures for high‐performance engineering applications.

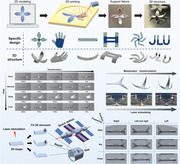

## 1 Introduction

4D printing is generally regarded as 3D printing of smart materials that can change shape or other properties with time under external stimuli including humidity,^[^ ^1^ ^]^ light,^[^ ^2^ ^]^ heat,^[^ ^3^ ^]^ electric fields,^[^ ^4^ ^]^ or magnetic fields.^[^ ^5^ ^]^ The materials used mainly include hydrogels,^[^ ^6^ ^]^ shape memory polymers,^[^ ^7^ ^]^ alloys,^[^ ^8^ ^]^ and liquid crystal elastomers.^[^ ^9^ ^]^ Among the above materials, a 4D printed sample based on polymer materials has a large deformation range, low mechanical strength, slow response speed, and low driving force.^[^ ^10^ ^]^ Meanwhile, the shape memory alloy represented by NiTi alloy has the advantages of good mechanical properties and a large driving force.^[^ ^8^, ^11^ ^]^ However, due to its shape change mechanism of phase transition, it is difficult to achieve large deformations. 4D printing materials are currently limited to polymers except for shape memory alloys, which narrows its applications in engineering fields. Therefore, it is of great significance to break through the restrictions of materials for 4D printing.

Laser powder bed fusion (LPBF) is one of the key additive manufacturing technologies, which melts metallic powders and solidifies to 3D part layer by layer.^[^ ^12^ ^]^ During the LPBF process, residual stresses are often generated due to the high cooling rates and large temperature gradients, which in turn cause processing defects such as delamination, cracks, and deformations in the formed part (Section S1, Supporting Information).^[^ ^13^ ^]^ The generation and distribution of stress is directly related to the scanning strategy (Figure S1, Supporting Information).^[^ ^13^, ^14^ ^]^ In any case, stress concentrations generally develop at the edges of the deposited layer and the interface between the deposited layer and the substrate, which affects the accuracy and mechanical strength of the forming part.^[^ ^13^, ^14^ ^]^ Therefore, increasing researches are focusing on avoiding or reducing thermal stress to improve the performance of samples.^[^ ^13^, ^14^, ^15^ ^]^

In contrast, the deformability caused by laser‐induced thermal stress can be controlled to reshape 3D printed structures, which can create a new 4D printing method. We propose the metallic 4D printing method based on the LPBF 3D printing technology, using the thermal stress generated by the laser to make the sample achieve shape morphing under the stimulus of laser scanning. This method uses laser as the stimulating heat source for metallic 4D printing to selectively induce thermal stress to convert the printed precursor into another tangible 3D structure, which not only provides a new strategy for structural transformation in 4D printing, but also makes the production process of 3D structures faster and more straightforward.

## 2 Results and Discussion

### 2.1 4D printing of Laser Stimulation

**Figure 1:**
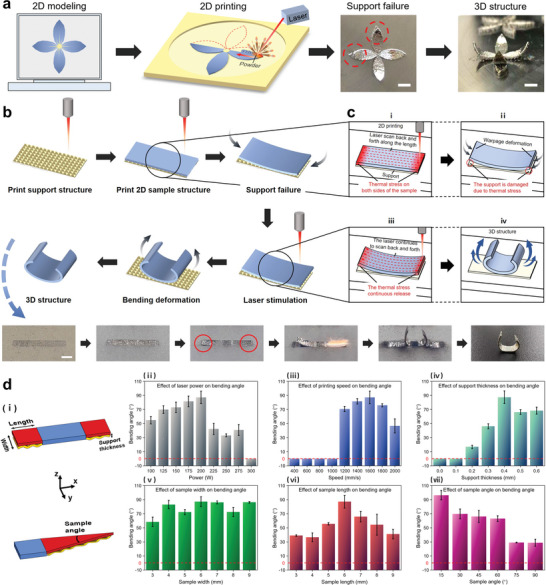
Laser powder bed fusion of metallic shape‐morphing samples. a) Schematic illustration of metallic 4D printing by pre‐setting laser thermal stress. Scale bars: 5 mm. b) Specific process of the metallic shape‐morphing samples triggered by laser. Scale bar: 5 mm. c) The effects of laser scanning strategies on the distribution of thermal stress: i) The samples are formed with the laser scanning tracks along the length direction (red arrow) to accumulate thermal stress (dark red gradation) for ii) damaging the support (red circle). iii) The laser continuously scans the sample to release the accumulated thermal stress and iv) finally causing the sample to gradually bend into the expected 3D structure. d) The influences of the ii–iv) processing parameters (laser power, scanning speed, support thickness) and v–vii) sample geometric parameters (sample width, length, and angle) on the shape‐morphing bending angle.

The metallic shape‐morphing objects were formed by laser powder bed fusion of 316L stainless steel. The thermal stresses are pre‐set in the selected areas during the layer‐by‐layer forming process, which can be released through the laser to morph objects into the expected structure. **Figure** **1**a–c shows the intuitive expression of the 4D printing by pre‐setting thermal stress. The 2D flower shape with supports is first modeled and printed (Figure 1a). Due to the support failure by laser scanning (red dotted circle), the petals bent forming the expected 3D flower structure. The detailed process is shown in Figure 1b and Movie S1 (Supporting Information). Taking the printing of a rectangle as an example, the support is printed first,^[^ ^16^ ^]^ and then the 2D sample precursor is printed. The thermal deformation of the sample at the beginning and the end of the laser scanning tracks is the largest.^[^ ^13^, ^14^ ^]^ Therefore, to control the support failure, the laser was set to print along the length of the sample, which causes a large amount of thermal stress to accumulate on both sides in this direction (Figure 1c‐i). When the accumulated thermal stress on both sides of the sample reaches the yield strength of the support,^[^ ^16^ ^]^ the support is broken, which causes the sample to slightly warp (Figure 1c‐ii).^[^ ^13b^ ^]^ Finally, the laser scans repeatedly along the length of the sample several times (Figure 1c‐iii). At this time, because the support has been damaged, each scan of the laser will release the thermal stress separately. Due to the continuous release of thermal stress, most of the two sides of the sample are separated from the support and gradually produce greater bending deformation,^[^ ^13b^ ^]^ which gradually transforms the sample from the initial 2D structure to the expected 3D structure (Figure 1c‐iv). By this method, printing some complex three‐dimensional structures becomes easier than before, and we no longer need to carry out complex operations such as multiple layer‐by‐layer overlay printing, printing difficult thick support and wire cutting post‐processing, which reduces the production time and makes the additive manufacturing more flexible. More importantly, it is possible to form shape‐morphing structures that are difficult to process using traditional 3D printing methods.

The achievable shape‐morphing bending angle of the sample represents an important parameter for evaluating the thermal stimulation performance, which is significantly affected by the processing parameters and the geometric parameters of the sample. Figure 1d‐ii–vii shows the effects of laser power, scanning speed, support thickness, sample width, sample length and sample angle on the shape‐morphing bending angle of the sample. Except for the sample angle using the triangular model for analysis, the other characterizations all use the rectangular model for analysis (Figure 1d‐i and Figure S2, Supporting Information). Based on previous experimental investigations (Figure S3, Supporting Information), it was found that under the premise of printing angle of 0°, when the laser power is 250 W and the printing speed is 1600 mm s^−1^, the upper and lower sides of the sample had the most serious warpages. It can be concluded that these process parameters are the substantial facts affecting the warpage of printed objects. Therefore, we explored the effects of these parameters on the bending angle of the samples with 15 printing layers. And the influences of support thickness, sample width, sample length and sample angle on the bending angle of the sample are also explored (Figures S4–S9, Supporting Information). It can be seen that when the processing parameters and geometric shape parameters are suitable, the bending angle of the rectangular sample can reach 87.08°, and the corresponding CT image is shown in Figure S10 (Supporting Information). When the geometric shape is triangular, the bending angle of the sample reaches a larger value of 96.3°. The specific reasons have been analyzed in Section S2 (Supporting Information). These characterizations can be used to determine the optimal parameters for programming the shape‐morphing of 4D printed structure.

### 2.2 Specific Design of 2D Precursor

**Figure 2:**
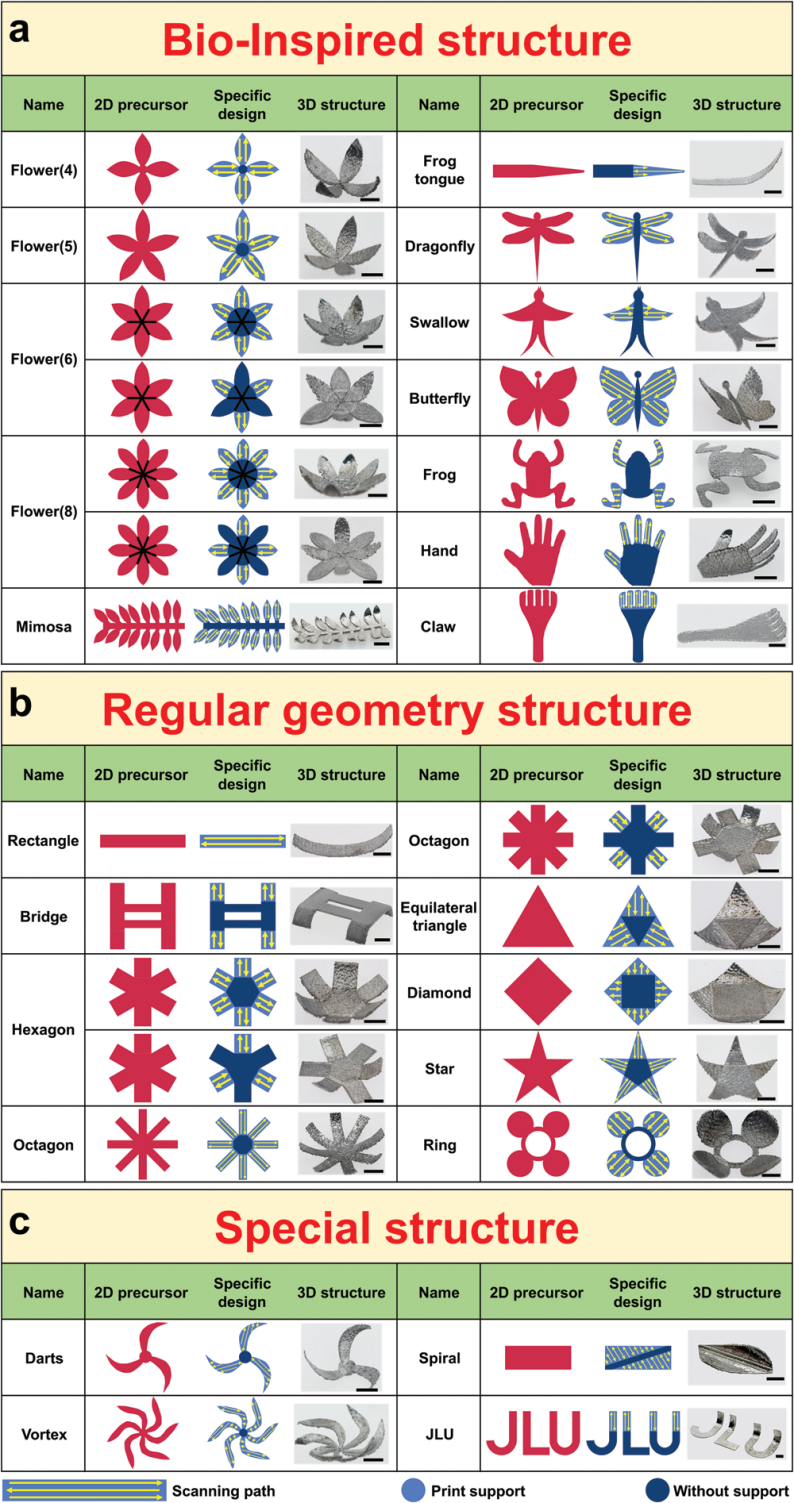
Experimental study of a series of 2D‐to‐3D structural transformations triggered by laser‐induced thermal stress. a) Fourteen types of bioinspired structures, b) ten types of regular geometric structures and c) four types of special structures. The yellow arrows represent the laser scanning paths, and the red, light blue and dark blue parts represent the 2D precursor and the parts with and without supports, respectively. Scale bars: 5 mm.

The specific process of metallic 4D printing is to convert the printed precursor based on the processing parameters programming into the desired 3D structure through laser stimulation. Therefore, the specific programming design of the 2D precursor is vital to obtain the desired 3D structure. It includes the segmentation method of the 2D precursor model, the programming of the laser parameters and support distribution. Among them, the laser scanning strategy has a significant impact on the distribution of thermal stress (Figure S1, Supporting Information),^[^ ^13^, ^14^ ^]^ which can be programmed to pre‐set strain on the selected area for shape deformation, combining with the design of 2D model segmentation and support distribution.^[^ ^16^ ^]^ Herein, the metallic 4D printing method can be further extended by designing 2D precursors to build various 3D structures with highly diversified geometric structures. **Figure** **2** summarizes the specific design of the print support distribution and the laser scanning strategy for 28 representative 2D precursors, as well as the final 3D structures, which can be divided into three categories: bioinspired structures, regular geometric structures and special structures. Each shape is marked with a name. Here, red represents the 2D precursor, the yellow arrows represent the laser scanning paths, and light blue and dark blue represent the parts with and without support, respectively. These specific designs of metallic 4D printing can strategically release thermal stress to convert the precursors into predetermined 3D structures.

In nature, the shape‐morphing movements of organisms can be seen everywhere, such as flowers, leaves and wings, etc. They will move on specific occasions to accomplish the corresponding purposes.^[^ ^1^, ^3^, ^10^ ^]^ Inspired by these biological systems, fourteen representative bioinspired metallic structures were printed (Figure 2a), through using laser stimulation to achieve the shape‐morphing of certain parts. The shape‐morphing structures are inspired by flowers (four, five, six and eight petals), mimosa, frog tongue, dragonfly, swallow, butterfly, frog, hand and claw.

The various 3D shape‐morphing structures deformed by the specific design of the two types of 2D precursors of the flowers (six and eight petals) demonstrate that the deformation can be controlled position‐specifically by adding supports or not, combined with the control of the thermal stress distribution by laser processing parameters programming, and then the specific structure and geometry of the sample can finally be formed and controlled. Specifically, when a part of the sample contains supports and the laser path is programmed to accumulate the thermal stress at the edge, this part will eventually release the thermal stress and deform. In contrast, due to the restraint of the substrate during forming, the printed part will not deform.

Figure 2b provides ten representative examples with regular geometric structures. The designed precursors are mainly composed of planes with different regular geometric shapes. The initial shapes are mainly 2D rectangles, triangles and circles, and the final 3D structures include rectangle, bridge, hexagon, octagon, equilateral triangle, diamond, star and ring.

Figure 2c provides four representative examples with special structures, including darts, vortex, spiral and “JLU” (Figure S11, Supporting Information). Different from regular geometric structures, special structures are composed of some unconventional special shapes, or formed by unconventional deformation mechanisms, or their shapes can represent some special meanings. Specifically, the 2D precursors of darts and vortex are composed of different numbers of crescents, spiral is a structural transformation completed by laser scanning angular characteristics, while “JLU” represents the Jilin University. The 3D structures obtained by the spiral in the above examples are different from the rectangle in the regular geometric structures, which proves that the same shape can be deformed into various 3D structures through programming the designs of 2D precursors (Figure S12, Supporting Information). The above 28 examples show that the unique combination of segmentation method, support design and laser parameters programming can make the metallic shape‐morphing structure more complex and changeable, which can further inspire the understanding and exploration of 4D printing applications.

### 2.3 Specific Process from 2D‐to‐3D

**Figure 3:**
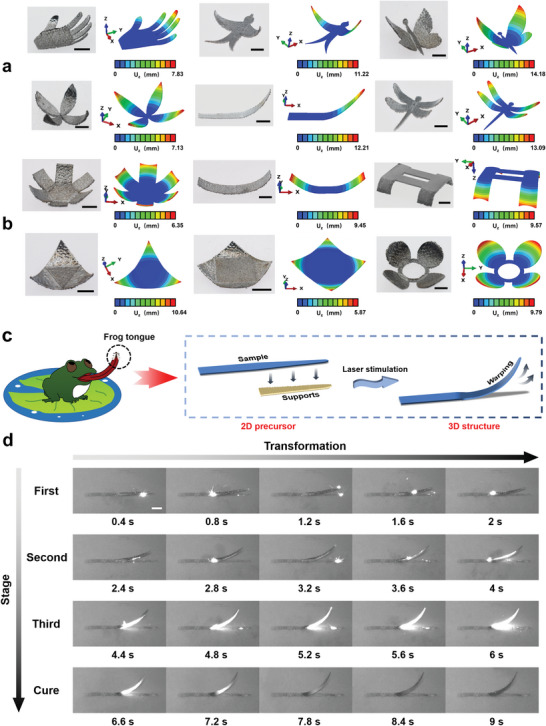
Shape‐morphing mechanism analyses of metallic 4D printing stimulated by laser. a) Shape‐morphing FEA results of hand, swallow, butterfly, flower, frog tongue, dragonfly in bioinspired structures, and b) hexagon, rectangle, bridge, triangle, diamond and ring in regular geometry structures. The color bar of the FEA result indicates the distribution of the displacement component U_Z_ (along the *Z* axis). Scale bars: 5 mm. c) Schematic illustration of the bending of the bioinspired frog tongue and d) the specific process of bending in single‐layer laser scanning. After three laser stimulations and cooling cycles, the bioinspired frog tongue gradually transforms from the 2D precursor to the 3D structure, and the bending angle is ≈85°. Scale bar: 5 mm.

To reveal the shape‐morphing mechanism of laser programmed metallic 4D printing, twelve samples were subjected to FEA, and the shape morphing process of the bioinspired frog tongue was presented to obtain a clearer understanding of laser stimulation (**Figure** **3**). In Figure 3a,b and Movie S2 (Supporting Information), we performed FEA of the thermally stimulated deformation on twelve representative 3D structures among the bioinspired structures and regular geometry structures (Section S3, Supporting Information).^[^ ^17^ ^]^ It can be seen that U_Z_ reaches the maximum at the edge of the deformed part (such as the fingers of the hand, the wings of the swallow and the butterfly, the sides of the rectangle and the hexagon, the corners of the triangle and the diamond, etc.) and reaches 0 on the base plane. This shows that the bending angle of the sample is the largest at the position where the support initially fails. The results of FEA are in good agreement with the experimental results, which proves the rationality and feasibility of the method and shows that it can be used as a useful design tool for thermally controlled and mechanically guided 3D assembly of complex shapes.

The specific process of the 2D‐to‐3D structural transformation of the biomimetic frog tongue is shown in Figure 3c,d and Movie S3 (Supporting Information). Specifically, the support of the tip of the 2D bioinspired frog tongue precursor was damaged by laser scanning during the processing, resulting in bending of the tip and finally performing the 2D‐to‐3D structural transformation (Figure 3c). The laser was set to scan along the length of the frog tongue, and the single layer scans continuously three times and the part stays for a period of time for cooling. The specific model parameters are given in Figure S13 (Supporting Information). The process of the single‐layer laser scanning deformation of the biomimetic frog tongue is shown in Figure 3d and Movie S3 (Supporting Information), which are divided into the first, second, and third stages of laser scanning and cooling stage. During the first laser scanning (0.4–2 s), it can be seen that after the support is damaged, the sample begins to gradually change from the 2D precursor to the 3D structure, and a slight warpage of ≈30° occurs. During the second laser scanning (2.4–4 s), the sample gradually bends upward while the degree of warpage increases, reaching ≈60°. This is because the body of tongue is printed directly without a support. When thermal stress is continuously released in the tip causing it to deform freely, the body will restrain the deformation, which triggers the tip to gradually change from warping to upward bending. In the third stage (4.4–6 s), the surface of the sample was burned due to the continuous scanning of the laser, which was more obvious at 5.2–6 s. At the same time, at 4.4–4.8 s, the sample only bent slightly and no longer deformed at 5.2–6 s, and the bending reached ≈75°. During the cooling stage (6.6–9 s), the burning on the surface of the sample gradually faded. Interestingly, the sample still bended upward slightly. There are two reasons to explain this phenomenon. First, because the sample has just undergone continuous laser scanning, the small amount of residual thermal stress remaining inside causing it to bend.^[^ ^13b,d^ ^]^ The second reason is uneven cooling. In the deformed sample, the shrinkage is small where the cooling is fast, and large where the cooling is slow.^[^ ^13d^ ^]^ In the 7.8–8.4 s image, it can be seen that the cooling rate of the lower side is slower than that of the upper side, so a certain degree of shrinkage occurs. Combining the above two reasons, the sample is eventually bent slightly. At 9 s, the cooling of the sample ends, and the bending angle reaches ≈85°. The sample is transformed from the initial 2D precursor to the 3D structure through the design of a laser scanning strategy and support distribution, achieving the bending effect of the frog tongue.

The above bioinspired frog tongue experiments illustrate that continuous laser scanning will deform the sample gradually. At the same time, it also affects the surface properties. In order to observe the surface changes of the sample more clearly, the single petal flower structure was printed and scanned by laser for 12 times after the support was damaged (Figure S14 and Movie S4, Supporting Information). The laser re‐scanning in this method is equivalent to in situ post‐processing of the surface via the laser remelting. The surface of the sample will be smoother and the mechanical properties will also be improved via the laser remelting process.^[^ ^18^ ^]^ The single petal flower structure has undergone large bending deformation through 12 times laser scanning. The surface quality has been improved, and the structure has not been damaged.

### 2.4 Laser Stimulation on Demand

**Figure 4:**
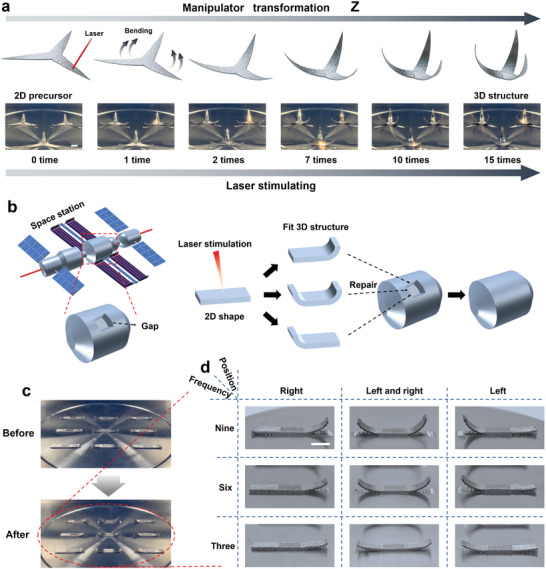
Application examples of metallic 4D printing stimulated by laser. a) The printed 2D manipulator precursor is bent by laser stimulation and then deformed into a 3D structure. Scale bar: 5 mm. b) The 2D part precursor can be bent on demand by laser stimulation to obtain a suitable 3D structure to repair irregular gaps, such as space station repair. c) The structural changes obtained by scanning the same part according to nine different laser scanning strategies. d) Nine specific laser scanning strategies and the 3D structures of the resulting parts. Scale bar: 5 mm.

In the field of traditional machining, operations such as the execution of industrial machines (robots, manipulators, etc.) and the manufacturing of parts have been extensively studied.^[^ ^19^ ^]^ The metallic 4D printing scheme introduced in this article shapes the sample by laser stimulation, which is easy to operate and can provide a new means using the above operations. **Figure** **4** shows two examples, including the control of the bending of the manipulator and the 3D irregular repair part by laser stimulation. In Figure 4a and Movie S5 (Supporting Information), bending of the manipulator by laser stimulation is achieved, and the printed 2D manipulator is deformed into the 3D structure (Figure S15, Supporting Information). This provides a new method for the bending operation of the metallic manipulator and increases its application value.

In today's aerospace field, damage to satellite parts or space stations is a very common problem.^[^ ^19d^ ^]^ Generally, the damage of parts is random, and the size and shape are irregular and not uniform, which often makes astronauts eager to remedy but cannot find suitable parts, and increases the risk of satellite malfunction. Metallic 4D printing of laser stimulation can be employed to control the shape transformation of the parts (Figure 4b–d), which can be used in space. As shown in Figure 4b, the 2D part precursor can be turned into fitting a 3D structure by laser stimulation to repair the gap. Specifically, the laser scanning strategy can be controlled according to the shape, size and angle of the required part to selectively stimulate the 2D part precursor to change it into a suitable 3D structure. The shape‐morphing structures obtained by stimulating the same part according to nine different laser scanning strategies are shown in Figure 4c,d and Movie S6 (Supporting Information). With the change in scanning frequency and scanning position, the parts can be controlled into 9 shapes with different bending degrees (Figure 4d), which proves that the specific shape of the part can be controlled by controlling the laser scanning strategy to finally obtain the expected 3D structure. At the same time, it is also evidenced that the method is applicable to other fields such as novel equipment and biomedical devices.

Compared with most materials used in 4D printing, such as polymers^[^ ^13^, ^17^ ^]^ and hydrogels,^[^ ^13^, ^17^ ^]^ the metal samples of 4D printing have higher mechanical properties (Figure S1 6and Movie S7, Supporting Information), which proves that this 4D printing method has a wide range of application value and potential in the engineering field. At the same time, the metallic 4D printing of laser stimulation allows the deformation of metallic 3D structures with a high degree of freedom. The shape‐morphing principle is to use laser as the stimulating heat source to strategically induce the internal thermal stress of the sample to achieve 2D‐to‐3D structural transformation, which proves that metallic 4D printing allows free‐style structural changes and is not limited to specific occasions. We only need to prepare a high‐power laser and program the laser parameters to temporal‐spatially shape the sample, which will make the production of 3D structures separate from the interior of the 3D printer and proceed in positions that are difficult to print using traditional 3D printing systems.

## 3 Conclusion

We have demonstrated a 4D printing pathway of common metallic materials relaying on the programming of processing parameters and scanning strategies. The method realizes metallic structural transformation by strategically inducing internal thermal stress of the sample in the printing process. This enables more metal materials to realize 4D printing of self‐deformation applications. It only needs to prepare a high‐power laser to stimulate the sample, which proves that this 4D printing method allows free‐style structural changes and is not limited to specific applications. The versatility of the programming route also provides an opportunity for more materials other than metal to be used in 4D printing. The methodology makes 4D printing more valuable and thus opens new avenues to create shape‐morphing 3D structures for high‐performance engineering applications. However, this concept is still in its infancy and being explored preliminarily. The service performances of metal 4D printed parts in engineering applications still need to be further investigated.

## 4 Experimental Section

### LPBF and 316L SS Powders

LPBF uses laser as the energy source and is specifically used as a method for printing metal parts.^[^ ^12a,b^ ^]^ The thermal stress generated via the laser can deform the printed sample.^[^ ^13b^ ^]^ The 316L ss as the basic material of the 2D precursor for capability demonstration is mainly due to the wide similarities of metals, 316L ss can provide a reference for most other metals,^[^ ^20^ ^]^ which explains the universality of this research. The gas atomized spherical 316L ss powders (Hanbang 3D Technology Co., Ltd.) were selected for the LPBF process as the basic material of the 2D precursor for capability demonstration. The powder particles were mainly spherical with a particle size distribution of 5 to 45 µm, and the average diameter was about 30 µm.

### Printing Process

The LPBF printer (Hanbang 3D Technology Co., Ltd.) equipped with a fiber laser (IPG Laser GmbH.) was used to prepare samples. Here, the laser wavelength was 1070 nm and the laser spot size was 55 µm. In the experimental investigation, in order to obtain a better bending effect, a laser power of 200 W and a printing speed of 1600 mm s^−1^ were used when printing all 2D precursors (Figure 1d). And the hatch distance (distance between scanning lines) was 50 µm, the layer thickness was 40 µm, the fill pattern type was stripe pattern and the path planning method was single fill. The scanning of contour was turned off, upper surface and lower surface, and only the scanning of inner surface was turned on. In the LPBF process, the molten pool was protected with inert argon, and the oxygen level was about 0.5%. The circular substrate of stainless steel was used. After the deformation of the sample was completed, they were cut from the substrate by wire cutting.

In the metallic 4D printing of laser stimulation, the support of the 2D precursor was an indispensable part. Here, according to the characterization result of Figure 1d, the thickness of all 2D precursor supports was set at 0.4 mm. In addition, the type of support was non‐entity. The laser power and scanning speed used to print the support were fixed. In order to make the support easier to be damaged, the laser power was 80 W and the scanning speed was 1200 mm s^−1^.

### Characterizing the Shape‐Morphing Procedure

The printing process of the bioinspired frog tongue was photographed by a USB3.0 micro industrial camera (Yingshi Technology Co., Ltd.) equipped with a 2 million 1/2" target surface industrial lens. Here, the parameters of the lens were: the pixel was 2 MP, the focal length was 4–12 mm, the target surface was 1/2″, and the aperture was F1.6‐C. The parameters of the camera were: the resolution was 640 × 480, the target surface was 1/4", the pixel size was 4.8 µm, the frame rate was 815 FPS, and the exposure time was 1000 us.

### Finite Element Analysis

The commercial software ABAQUS CAE was used to perform finite element analysis on the warping simulation of the sample to show the mechanism of the deformation process (Figure 3a,b and Movie S2, Supporting Information). Due to the high computational cost of the track‐by‐track deposition in FEM (finite element method), quantitatively simulating the whole manufacturing process of each case was unrealistic. As the parts were thin‐wall structure, the strain level on the free surface and near base plane were quite different. Thus, the warping was modeled based on the inherent strain method.^[^ ^13^, ^17^ ^]^

## Conflict of Interest

The authors declare no conflict of interest.

## Author Contributions

G.L., W.W., Y.Z., Q.L.: Conceptualization; G.L., Y.Z., F.C.: Methodology; G.L., Y.Z., W.W., X.L., A.Z.: Investigation; G.L., Y.Z., W.W., F.C.: Visualization; G.L., W.W., L.R.: Funding acquisition; G.L., W.W., Q.L.: Project administration; G.L., W.W., Q.L., J.Z., L.R.: Supervision; G.L., Y.Z., W.W., Q.L.: Writing—original draft; G.L., Y.Z., W.W., X.L., A.Z., J.Y.H.F., L.R., Q.L., F.C., X.L., J.Z.: Writing—review and editing.

## Supporting information

Supporting Information

Supplemental Movie 1

Supplemental Movie 2

Supplemental Movie 3

Supplemental Movie 4

Supplemental Movie 5

Supplemental Movie 6

Supplemental Movie 7

## Data Availability

The data that support the findings of this study are available from the corresponding author upon reasonable request.
