# Supplementary material for: Metallic 4D Printing of Laser Stimulation
Source: Adv Sci (Weinh). 2023 Jan 22;10(12):2206486. doi: 10.1002/advs.202206486 (PMC10131821; doi:10.1002/advs.202206486)
Supplement: Supplementary file 1 — Supporting Information [file ADVS-10-2206486-s005.pdf]

ADVANCED SCIENCE
Open Access

Supporting Information

for *Adv. Sci.*, DOI 10.1002/advs.202206486

Metallic 4D Printing of Laser Stimulation

*Wenzheng Wu, Yiming Zhou, Qingping Liu, Luquan Ren, Fan Chen, Jerry Ying Hsi Fuh, Aodu Zheng, Xuechao Li, Ji Zhao and Guiwei Li**

Supporting Information

Metallic 4D printing of laser stimulation

*Wenzheng Wu, Yiming Zhou, Qingping Liu, Fan Chen, Jerry Ying Hsi Fuh, Aodu Zheng, Xuechao Li, Luquan Ren, Ji Zhao, Guiwei Li**

Supplementary Text

S1. The thermal stress during LPBF forming process

Cracks are prone to occur during the LPBF forming process, and the occurrence of cracks is also a reason for the porosity of the LPBF formed parts. This is because LPBF is a rapid melting-solidification process, and the melt has a higher temperature gradient and cooling rate. The process occurs instantaneously in a short period of time, which will generate greater thermal stress. The thermal stress of LPBF is caused by the inconsistent thermal expansion and contraction deformation trends of various parts when the laser heat source acts on the metal. During the melting process, since the LPBF molten pool instantly rises to a high temperature, the molten pool and the areas with higher temperatures around the molten pool tend to expand, while the area far away from the molten pool has a low temperature and has no expansion trend. Due to the mutual restraint of the two parts, the position of the molten pool will be restrained by compressive stress, and the position away from the molten pool will receive tensile stress. The melt gradually shrinks during the cooling. On the contrary, the solidified part of the melt receives tensile stress, while the part away from the melt receives compressive stress. The accumulated stress is finally released in the form of cracks. It can be seen that the uneven heating of the LPBF process is the main cause of thermal stress.[1]

S2. The influence of processing parameters on the bending angle (Figure 1d (ii-vii))

From the analysis of the printing process, for a given geometric shape and size, the bending angle can be controlled by the process parameters. **(ii) (iii)** Laser power and scanning speed are related to laser energy density, which can be expressed as：

$$\rho = \frac{P}{tvh} \tag{1}$$

Where ρ is the laser energy density, P is the laser power, t is the layer thickness, v is the scanning speed, and h is the hatch distance.

When the laser power is increased (100-200 W) or the scanning speed is decreased (2000-1600 mm/s) within a certain range, the laser energy density will increase, which will

lead to an increase in the bending angle of the sample.[2] However, when the laser energy density is high enough (200-300 W, 1600-400 mm/s), the bending angle will be decreased until no bending occurs. Because as the energy density increases, more powder is melting, which will reduce the porosity of the sample and increases the support strength, and makes it difficult for thermal stress to separate them.[3] **(iv)** The support thickness has a direct impact on the bending angle. When the support thickness is smaller (0.4-0 mm), the substrate has a higher restraint on the support, and when the support thickness is higher (0.4-0.6 mm), the support strength will be increased and the support will not be easily damaged. Both of the above will reduce the bending angle.[4]

From the analysis of the properties of the sample itself, for a given printing process, the bending angle can be controlled by the size and geometric shape. **(v)** The influence of the sample width on the bending angle is not obvious (3-9 mm). The support area will increase with the increase of the sample width, which leads to an increase in the support strength.[4] At the same time, the laser will scan the surface for more time and generates more thermal stress, which offsets the above effect. **(vi)** Conversely, the sample length has a significant effect on the bending angle. If the length is too short (3-6 mm), the displacement of the sample along the vertical direction will become more difficult. If the length is too long (6-9 mm), the area of the support along the length will increase. Both of the above will eventually decrease the bending angle.[4] **(vii)** It can be seen that the bending angle is inversely proportional to the sample angle (15-90 °). Because when the length of the bottom side is constant, the area of the support around the corner becomes larger with the increase of the sample angle, which makes it difficult for the support to be damaged.[4]

S3. Finite element analysis

The finite element analysis is performed on the warping simulation of the sample to show the mechanism of the deformation process. (Figure 3a,b and Movie S2, Supporting Information). Due to the high computational cost of the track-by-track deposition in FEM (finite element method), quantitatively simulating the whole manufacturing process of each case is unrealistic. As the parts are thin-wall structure, the strain level on the free surface and near base plane are quite different. Thus, the warping is modelled based on the inherent strain method.

The inherent strain can be expressed mathematically as： [5]

$$\varepsilon^{In} = \varepsilon_{t1}^{Plastic} + \varepsilon_{t1}^{Elastic} - \varepsilon_{t2}^{Elastic} \tag{2}$$

Which can also be rearranged as：

$$\varepsilon^{In} = \varepsilon_{t1}^{Total} - \varepsilon_{t2}^{Elastic}$$
(3)

Where t_1 and t_2 represent the time corresponding to the intermediate and steady state for the point of interest in an AM process, respectively. $\varepsilon_{t1}^{Plastic}$、$\varepsilon_{t1}^{Elastic}$ and ε_{t1}^{Total} represent the plastic strain, elastic strain and total mechanical strain in the intermediate state, respectively. $\varepsilon_{t2}^{Elastic}$ represents the elastic strain in steady state.[5]

Since this simulation aims to qualitatively analyze the deformation trend, the specific history of mechanical strain calculation appears to be unnecessary. By assigning different inherent strain values into the elements of the parts according to its layers, the part distortion can be simulated. Due to the continuous scanning of the laser, the attribution of the inherent strain is changed from tension to compression, and from top surface to the base ground. For each case, the agglomeration approach is utilized to merge several layers together as a whole block with the attribution of different strains assigned to different regions according to their position. In this way the whole part is separated into 4 different blocks and the orthotropic base inherent strain component is set to be (-0.0045, -0.0045, 0.008).[5] Normally, due to the faster cooling rate on the top surface and energy accumulation in the lower layers, the strain state should be tensive on the top and compressive at the bottom.[6] Therefore, the coefficients for the 4 layers are chosen to be 0.03, 0.01, -0.01, -0.03 from top to bottom. According to reference，[5] the way to assign the inherent strains are through the equations for the thermal strains ε_j^{Th} calculation in j^{th} direction：[5]

$$\varepsilon_j^{Th} = \alpha_j \Delta T, j = x, y, z$$
(4)

Where α_j is the equivalent thermal expansion coefficient, ΔT is the temperature change set to be unity in this work.

Noted: the young’s modulus and Poisson’s ratio does affect the distortion, since the strain is determined by the attribution, but they will affect the stress level. This work only focuses on the distortion.

Supplementary Figures

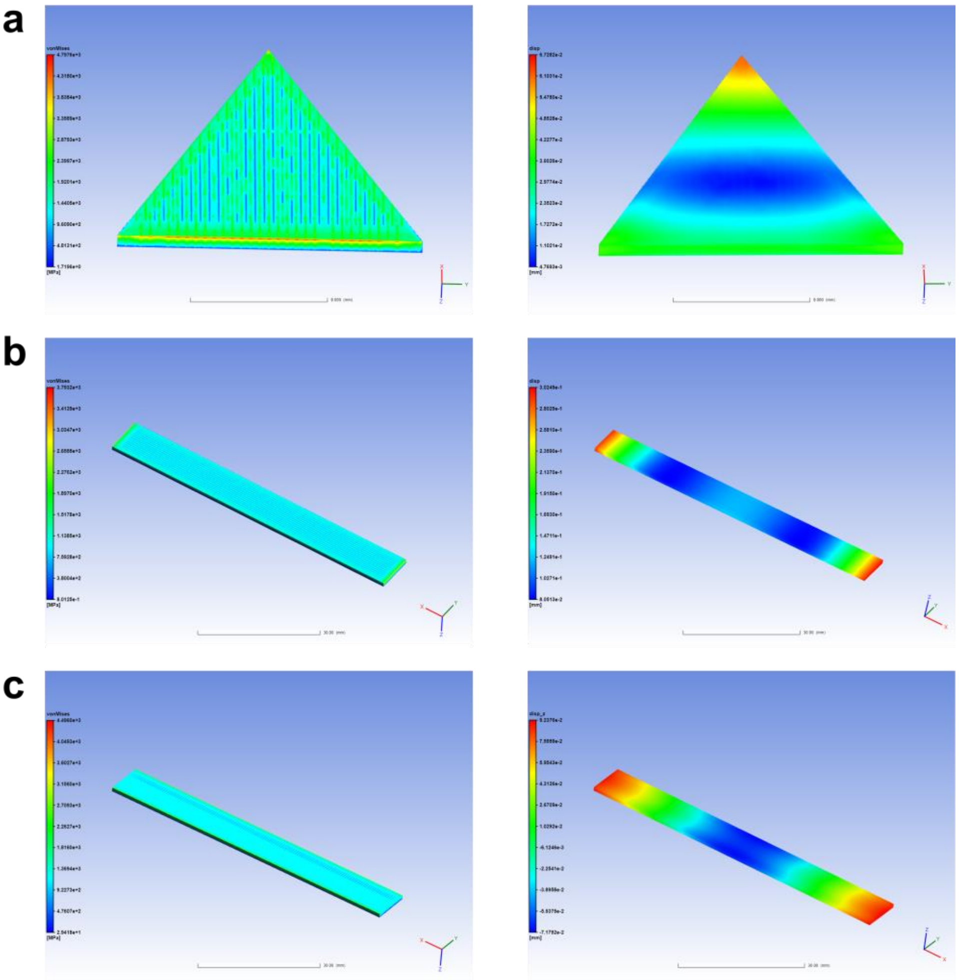

Figure S1. FEA analysis results of stress and displacement for the base model. The equilateral triangle sample with the laser scans along the X direction (a), and the two rectangular samples with the laser scans along the X (b) and Y (c) directions.

Discussion:

The laser scanning strategy has a direct effect on the residual stress and deformation of the sample.[1-2, 7] As shown in the triangular sample (a), the laser scanning along the X direction causes the maximum stress on both sides of the sample along the X direction, and also has a large displacement at the corners. As shown in the rectangular samples (b and c), when the laser scanning along the X direction, that is, along the length direction, the stress on both sides of the sample in the X direction is the largest and has a large displacement; when the laser scanning along the Y direction, that is, perpendicular to the length direction, the stress on both sides of the sample in the Y direction is the largest and there is a small displacement on both sides in the X direction. Therefore, it can be seen from the above simulation that the residual stress of the sample is the largest on both sides of the laser scanning path, and the sample will also be deformed greatly in this direction.

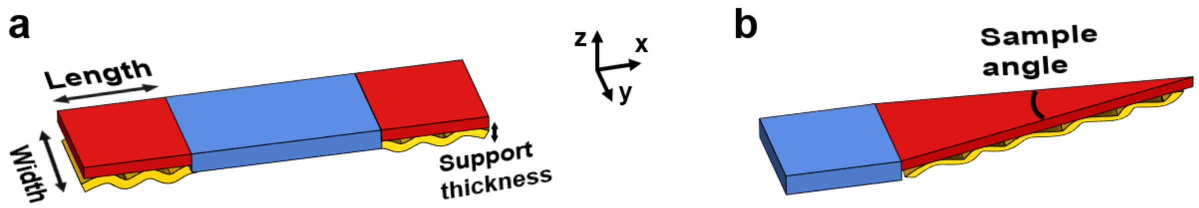

Figure S2. Rectangular(a) and triangular(b) models for characterization (Figure 1d). The blue area is the part directly printed, and red is the part printed with support. The formula for calculating the bending angle θ of the rectangular model: $\theta = \frac{\theta_1+\theta_2}{2}$, where θ_1 and θ_2 represent the bending angles of the left and right sides, respectively. When printing the triangle shape, keep the length of the bottom side unchanged, and change the height of the sample to change the angle of the sample. The scanning direction of the laser is along the X axis.

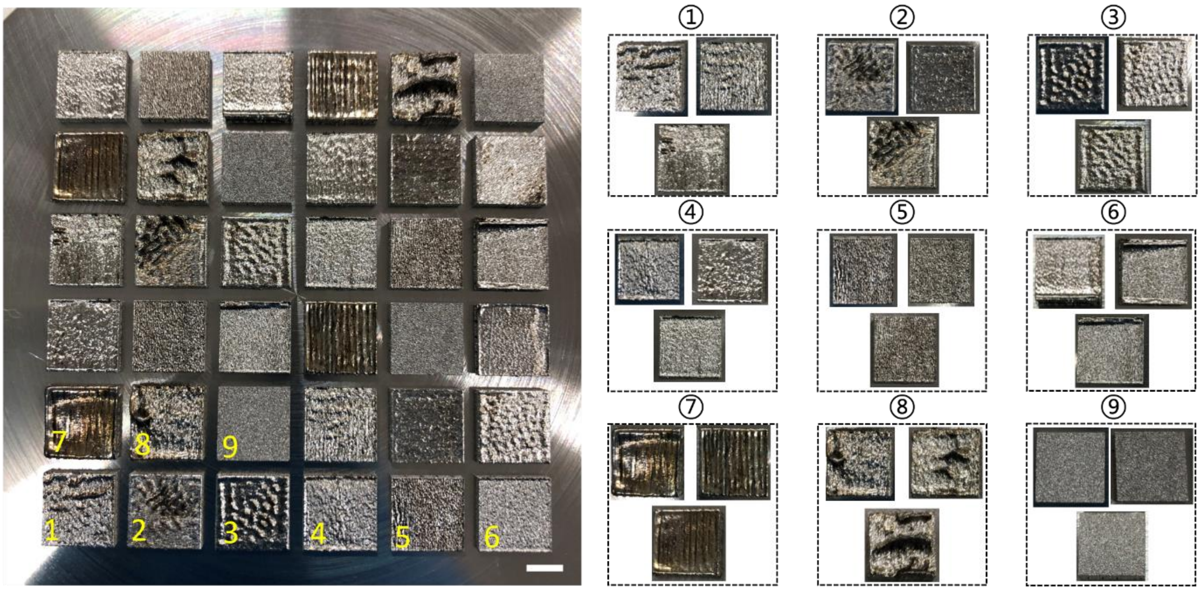

Number（1-9）	1	2	3	4	5	6	7	8	9
Power（W）	250	250	250	450	50	250	250	250	50
Speed（mm/s）	900	900	900	1600	200	1600	200	200	900
Angle（°）	0	90	37	0	0	0	0	0	0

Figure S3. Macroscopic analysis with different laser power, scanning speed, and printing angle.

There are nine groups in total, with four printed samples in each group and three of them are used for analysis. The support thickness, sample width, sample length and printing thickness are fixed, which are 0.4 mm, 10 mm, 10 mm and 0.6 mm, respectively. Scale bar: 5 mm.

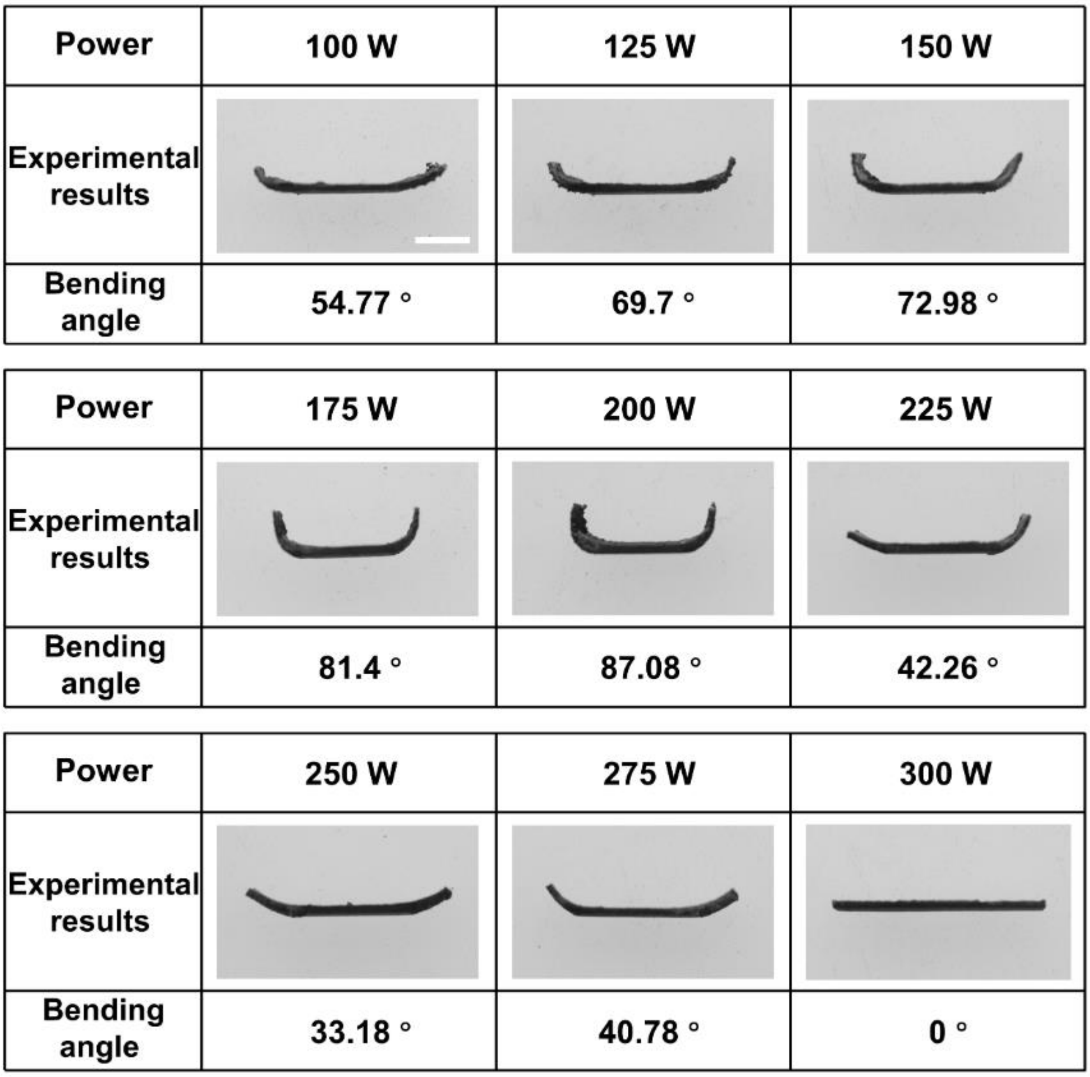

Figure S4. Experimental results of the influence of different laser power on the bending angle.

Here, the scanning speed, support thickness, sample width, sample length and sample thickness are fixed, which are 1600 mm/s, 0.4 mm, 6 mm, 6 mm and 0.6 mm, respectively. Scale bar: 5 mm.

Speed	400 mm/s	600 mm/s	800 mm/s
Experimental results	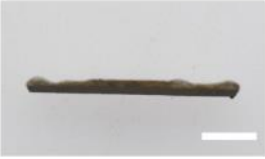	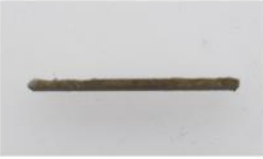	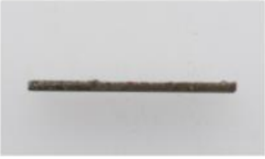
Bending angle	0 °	0 °	0 °

Speed	1000 mm/s	1200 mm/s	1400 mm/s
Experimental results	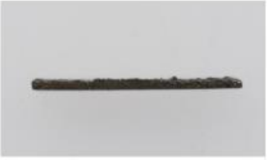	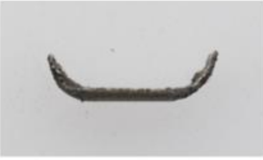	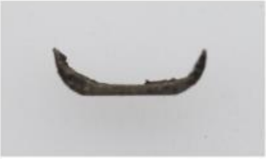
Bending angle	0 °	70.73 °	81.68 °

Speed	1600 mm/s	1800 mm/s	2000 mm/s
Experimental results	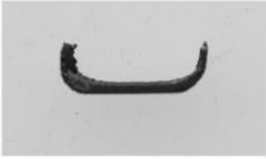	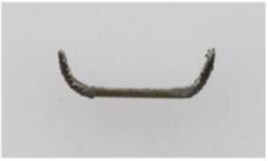	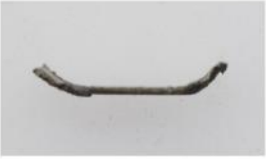
Bending angle	87.08 °	76.07 °	46.79 °

Figure S5. Experimental results of the influence of different scanning speed on the bending angle. Here, the laser power, support thickness, sample width, sample length and sample thickness are fixed, which are 200 W, 0.4 mm, 6 mm, 6 mm and 0.6 mm, respectively. Scale bar: 5 mm.

Support thickness	0 mm	0.1 mm	0.2 mm
Experimental results	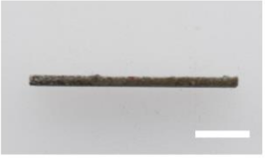	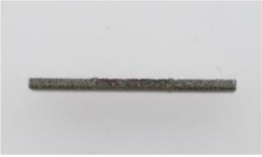	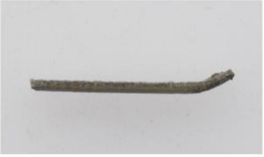
Bending angle	0 °	0 °	17 °

Support thickness	0.3 mm	0.4 mm	0.5 mm
Experimental results	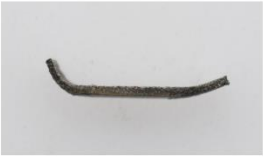	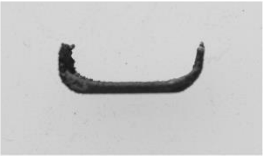	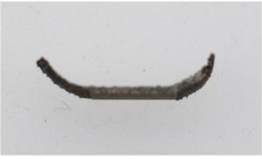
Bending angle	45.83 °	87.08 °	66.18 °

Support thickness	0.6 mm
Experimental results	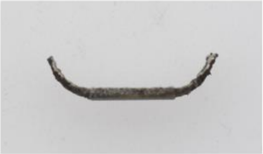
Bending angle	68.05 °

Figure S6. Experimental results of the influence of different support thickness on the bending angle. Here, the laser power, scanning speed, sample width, sample length and sample thickness are fixed, which are 200 W, 1600 mm/s, 6 mm, 6 mm and 0.6 mm, respectively. Scale bar: 5 mm.

Sample width	3 mm	4 mm	5 mm
Experimental results	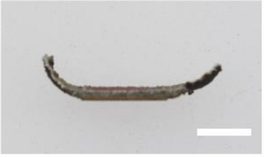	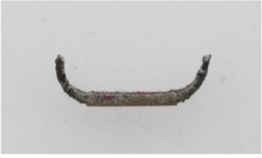	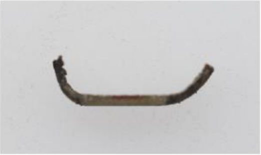
Bending angle	58.4 °	82.93 °	72.27 °

Sample width	6 mm	7 mm	8 mm
Experimental results	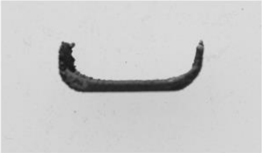	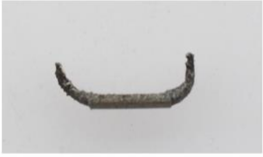	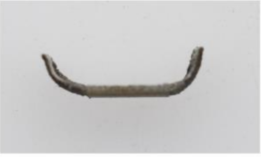
Bending angle	87.08 °	86.09 °	72.46 °

Sample width	9 mm
Experimental results	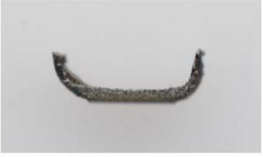
Bending angle	86.14 °

Figure S7. Experimental results of the influence of different sample width on the bending angle. Here, the laser power, scanning speed, support thickness, sample length and sample thickness are fixed, which are 200 W, 1600 mm/s, 0.4 mm, 6 mm and 0.6 mm, respectively. Scale bar: 5 mm.

Sample length	3 mm	4 mm	5 mm
Experimental results	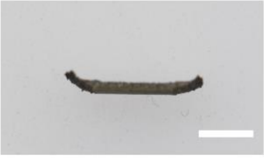	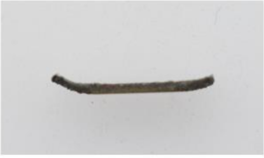	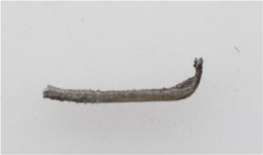
Bending angle	38.98 °	36.49 °	54.3 °

Sample length	6 mm	7 mm	8 mm
Experimental results	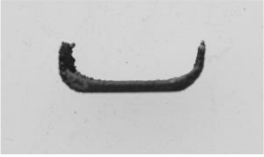	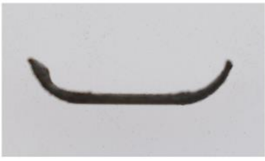	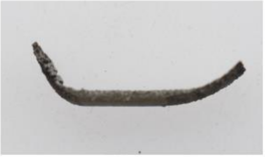
Bending angle	87.08 °	65.85 °	54.42 °

Sample length	9 mm
Experimental results	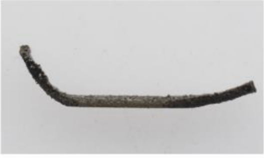
Bending angle	41.09 °

Figure S8. Experimental results of the influence of different sample length on the bending angle. Here, the laser power, scanning speed, support thickness, sample width and sample thickness are fixed, which are 200 W, 1600 mm/s, 0.4 mm, 6 mm and 0.6 mm, respectively. Scale bar: 5 mm.

Sample angle	15 °	30 °	45 °
Experimental results	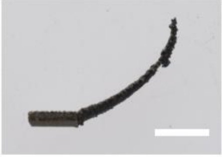	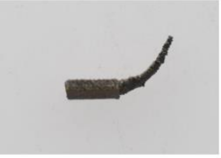	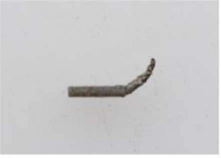
Bending angle	96.31 °	69.72 °	66.08 °

Sample angle	60 °	75 °	90 °
Experimental results	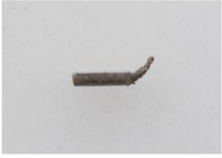	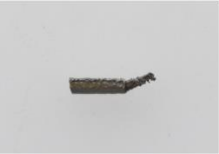	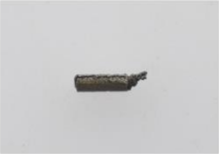
Bending angle	63.01 °	29.05 °	28.73 °

Figure S9. Experimental results of the influence of different sample angle on the bending angle. Here, the laser power, scanning speed, support thickness, and sample thickness are fixed, which are 200 W, 1600 mm/s, 0.4 mm and 0.6 mm, respectively. Scale bar: 5 mm.

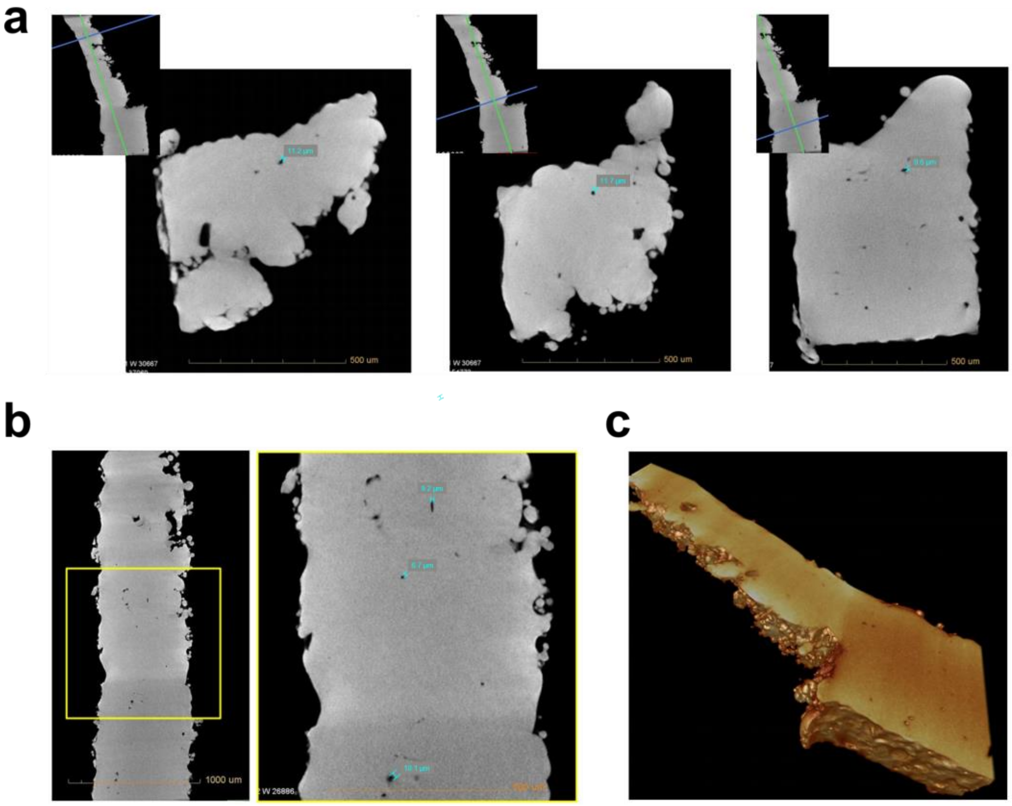

Figure S10. CT image of the rectangular sample after shape morphing (P: 200 W, v: 1600 mm/s). (a) Virtual slices images of the main deformation part, and the blue line in the upper left corner is the section position. (b) Virtual slice image of the green line section position in (a), and the right figure is the enlargement of the region of interest. (c) 3D rendered image of the main part next to the support layer. (The voxel resolution is 1.3 μm)

Discussion:

Figure S10 (a, b) presents the cross-sections of the processed sample, and the pore structure and size in the deformation area and undeformation area. There is no significant difference in the pores formed by processing defects in different areas. In particular, there are no defects such as cracks around the pores in the deformation zone. As shown in Figure S10 (c), the surface of the sample next to the support layer is kind of rough. However, there is no crack propagating to the sample on the supporting fracture surface, which illustrates that the surface bonded to the support has little effect on the main body of the deformed sample.

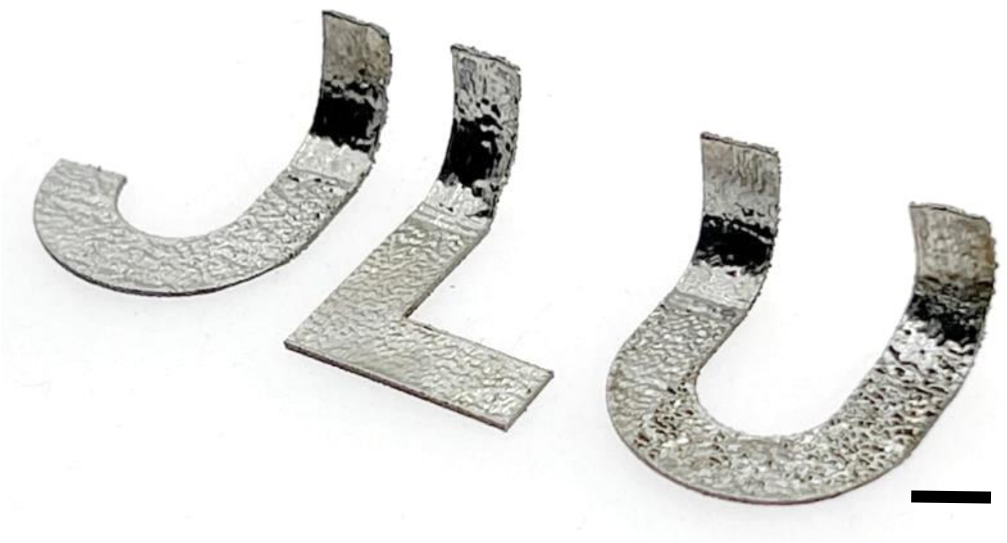

Figure S11. The 3D structure of the "JLU". Scale bar: 5 mm.

Discussion: This method can be used to print some special structures, such as the English abbreviation of the Jilin University ——"JLU". As shown in Figure S11, the designed 2D "JLU" precursor is finally transformed into a 3D structure with a certain bending angle by laser stimulation, which can stand on the desktop.

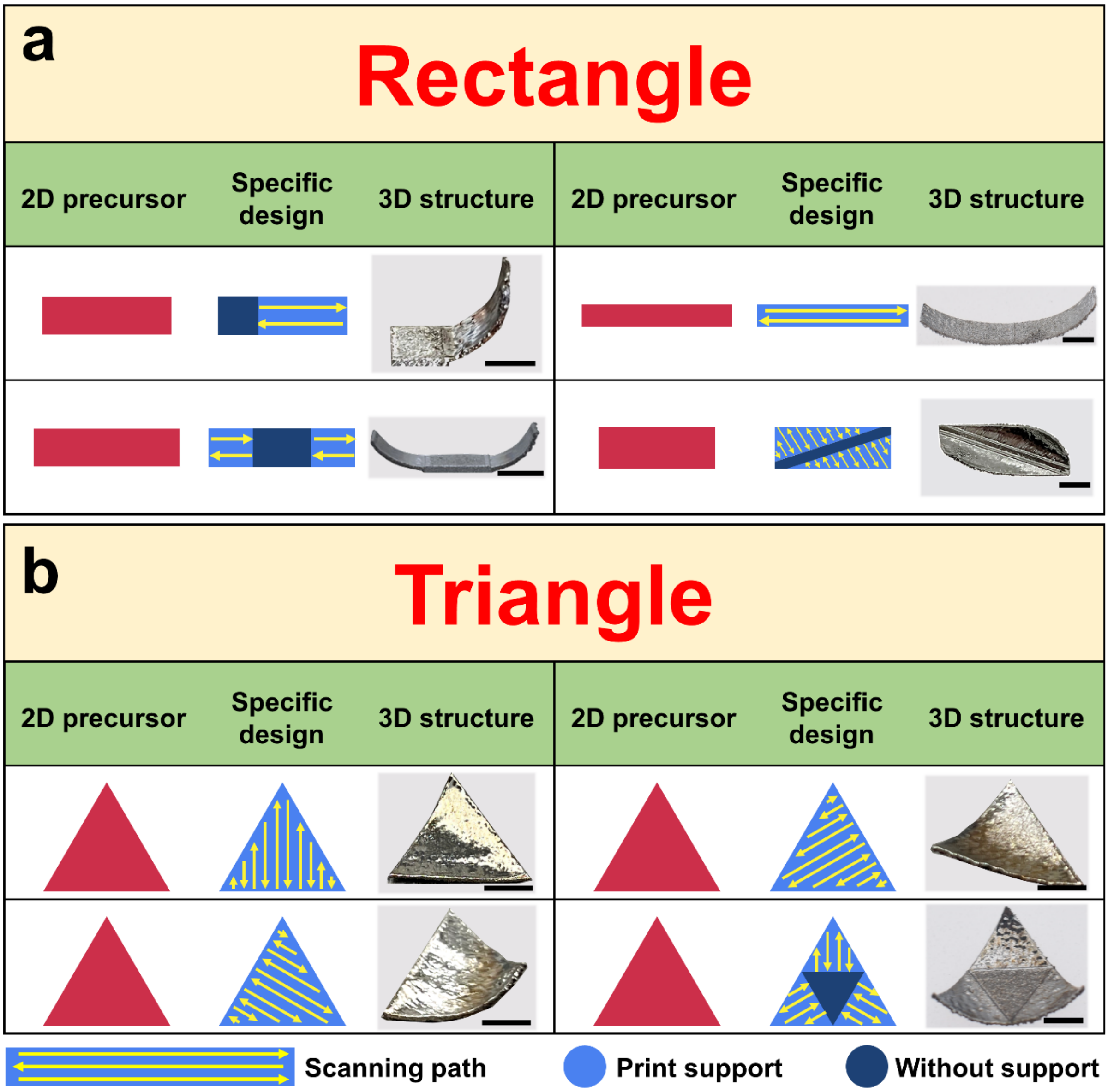

Figure S12. Specific designs of four rectangular (a) and triangular (b) 2D precursors and their shape-morphing 3D structures. Scale bars: 5 mm.

Discussion:

As for the same 2D precursor shape, different specific designs can generate different structural transformation effects. As shown in Figure S12, when the 2D precursor models are all (a) rectangular or (b) triangular, the specific designs of the four 2D precursors can result in distinct 3D structures with the laser stimulation.

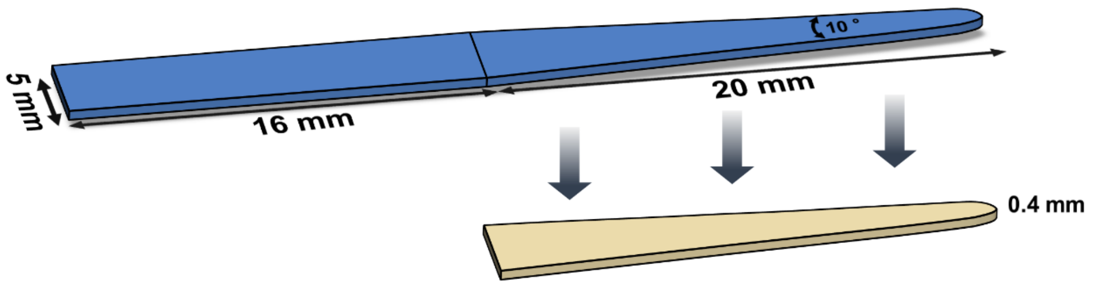

Figure S13. Model and process parameters of the bioinspired frog tongue (Figure 3c). Here, the overall length is 36 mm, and the left part is rectangular, 16 mm and printed directly; the right part is approximately triangular, 20 mm and printed with support. According to the results in Figure 1d, set the laser power is 200 W, the scanning speed is 1600 mm/s, the sample width is 5 mm, the sample angle is 10 °, and the support thickness is 0.4 mm.

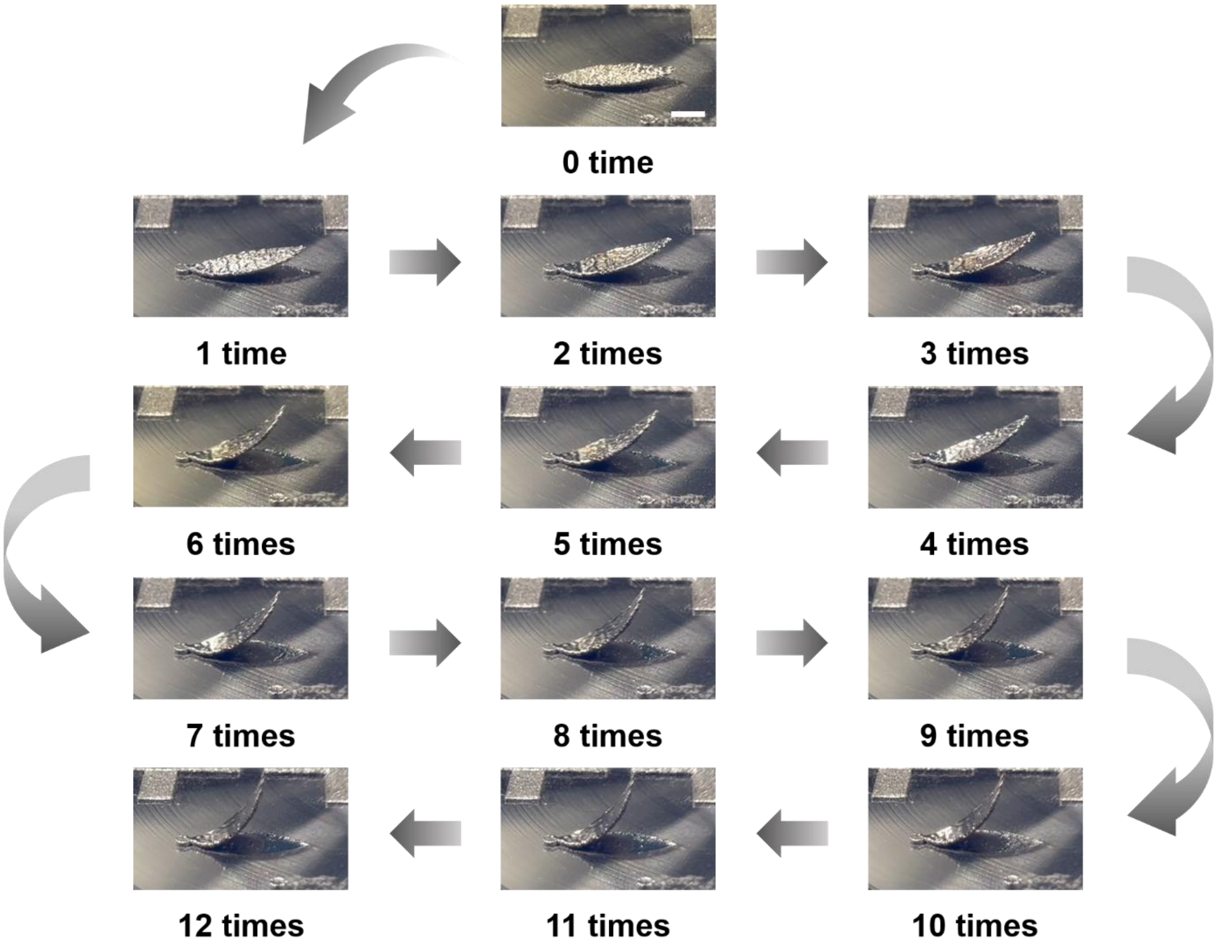

Figure S14. The effect of stimulation times on the surface of the single petal flower structure (P: 200 W, v: 1600 mm/s). Scale bar: 5 mm

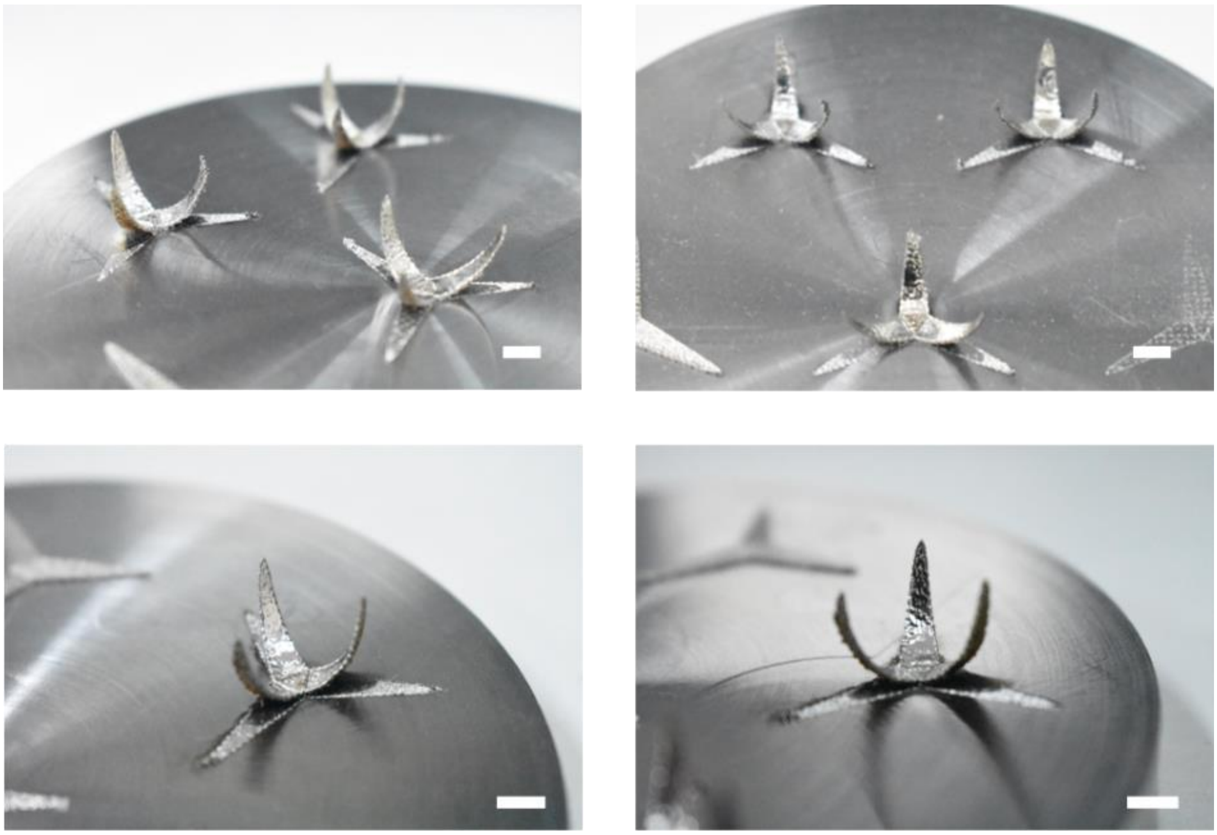

Figure S15. The 3D structure of the manipulators (Figure 4a). Scale bars: 5 mm.

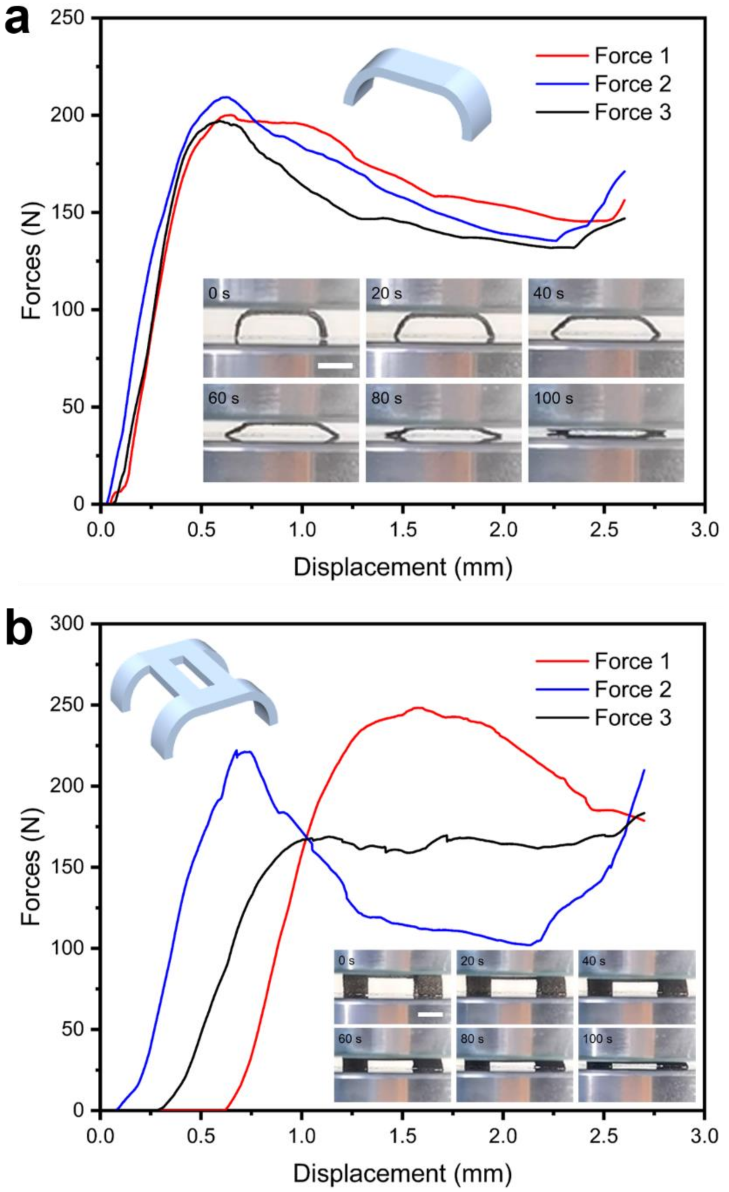

Figure S16. Compression force-displacement curves of (a) rectangular and (b) bridge structures. The compression speed is 1 mm/min, and the process parameters used for the two structures are the same: the laser power is 200 W, the scanning speed is 1600 mm/s, and the sample thickness is 0.6 mm. The maximum compressive force that the rectangular structure can bear is 202.137 ± 6.356 N, and the maximum compressive force that the bridge structure can bear is 212.867 ± 40.739 N. Scale bars: 5 mm.

Supplementary Movies

Movie S1: Process of the metallic shape-morphing samples.

Movie S2: Shape-morphing FEA results of 12 structures.

Movie S3: Process of the laser stimulation of bioinspired frog tongue.

Movie S4. Effect of the 12 times laser scanning.

Movie S5: Process of the laser stimulation of 2D manipulator.

Movie S6: Various effects of nine printing strategies on samples.

Movie S7: Compression test of rectangular and bridge structures.

References

[1] B. Cheng, S. Shrestha, K. Chou, *Additive Manufacturing* **2016**, 12, 240.
[2] a)Y. Lu, S. Wu, Y. Gan, T. Huang, C. Yang, J. Lin, J. Lin, *Optics and Laser Technology* **2015**, 75, 197; b)L. D. Bobbio, S. Qin, A. Dunbar, P. Michaleris, A. M. Beese, *Additive Manufacturing* **2017**, 14, 60.
[3] a)R. Li, J. Liu, Y. Shi, L. Wang, W. Jiang, *International Journal of Advanced Manufacturing Technology* **2012**, 59, 1025; b)L.-E. Loh, C.-K. Chua, W.-Y. Yeong, J. Song, M. Mapar, S.-L. Sing, Z.-H. Liu, D.-Q. Zhang, *International Journal of Heat and Mass Transfer* **2015**, 80, 288.
[4] a)Z. Zhang, C. Wu, T. Li, K. Liang, Y. Cao, *Rapid Prototyping Journal* **2018**, 24, 764; b)L. Mugwagwa, D. Dimitrov, S. Matope, I. Yadroitsev, presented at *15th Global Conference on Sustainable Manufacturing (GCSM)*, Technion Inst Technol, Haifa, ISRAEL, 2018 Sep 25-27, **2017**.
[5] X. Liang, L. Cheng, Q. Chen, Q. Yang, A. C. To, *Additive Manufacturing* **2018**, 23, 471.
[6] Y. Liu, Y. Yang, D. Wang, *International Journal of Advanced Manufacturing Technology* **2016**, 87, 647.
[7] a)T. Larimian, M. Kannan, D. Grzesiak, B. AlMangour, T. Borkar, *Materials Science and Engineering a-Structural Materials Properties Microstructure and Processing* **2020**, 770, 138455; b)L. Parry, I. A. Ashcroft, R. D. Wildman, *Additive Manufacturing* **2016**, 12, 1.
